# The ozone climate penalty, NAAQS attainment, and health equity along the Colorado Front Range

**DOI:** 10.1038/s41370-021-00375-9

**Published:** 2021-09-10

**Authors:** James L. Crooks, Rachel Licker, Adrienne L. Hollis, Brenda Ekwurzel

**Affiliations:** 1 National Jewish Health, Denver, CO USA; 2 Colorado School of Public Health, Aurora, CO USA; 3 Union of Concerned Scientists, Washington, DC USA

**Keywords:** Ozone, Climate penalty, Climate change, NAAQS, Environmental justice, Colorado

## Abstract

**Background:**

While ozone levels in the USA have decreased since the 1980s, the Denver Metro North Front Range (DMNFR) region remains in nonattainment of the National Ambient Air Quality Standard (NAAQS).

**Objective:**

To estimate the warm season ozone climate penalty to characterize its impact on Colorado Front Range NAAQS attainment and health equity.

**Methods:**

May to October ozone concentrations were estimated using spatio-temporal land-use regression models accounting for climate and weather patterns. The ozone climate penalty was defined as the difference between the 2010s concentrations and concentrations predicted using daily 2010s weather adjusted to match the 1950s climate, holding constant other factors affecting ozone formation.

**Results:**

The ozone climate penalty was 0.5–1.0 ppb for 8-h max ozone concentrations. The highest penalty was around major urban centers and later in the summer. The penalty was positively associated with census tract-level percentage of Hispanic/Latino residents, children living within 100–200% of the federal poverty level, and residents with asthma, diabetes, fair or poor health status, or lacking health insurance.

**Significance:**

The penalty increased the DMNFR ozone NAAQS design values, delaying extrapolated future attainment of the 2008 and 2015 ozone standards by approximately 2 years each, to 2025 and 2035, respectively.

## Abstract (web summary)

The ozone climate penalty in parts per billion (ppb) in the Denver Metro North Front Range region, with nonattainment areas located within the gray box. The ozone climate penalty was defined as the difference between ground-level ozone concentrations in the 2010s and concentrations predicted using daily 2010s weather adjusted to match the 1950s climate, holding constant other factors affecting ozone formation.

## Graphical abstract

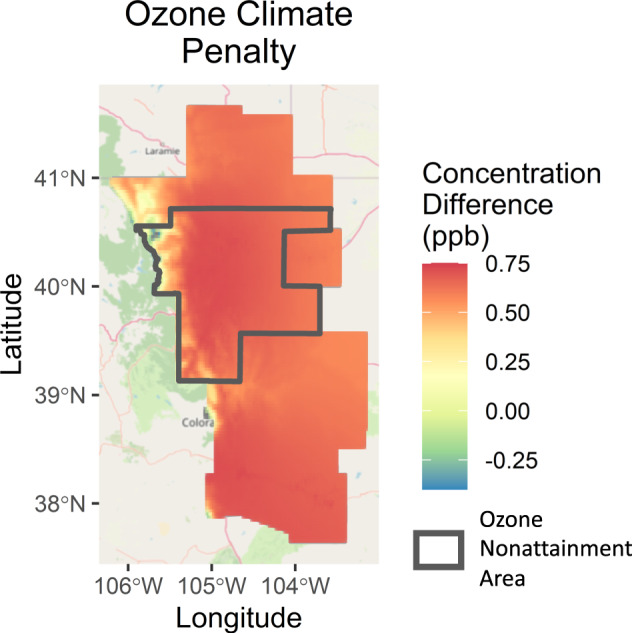

## Introduction

Ground-level ozone is a well-known respiratory irritant and component of smog. Ozone levels above US Environmental Protection Agency (EPA) standard are causally linked to a number of acute health effects, including eye and nose irritation, exacerbations of chronic respiratory diseases like asthma and chronic obstructive pulmonary disease (COPD), and adverse effects on lung function [1]. In addition, both short- and long-term exposures to ozone at concentrations below the current regulatory standards are associated with increased deaths from both respiratory and cardiovascular disease.

Ozone in the lower troposphere is produced through photochemical reactions with precursor emissions of volatile organic compounds (VOCs) and nitrogen oxides (NOx), which increase in the presence of heat [2]. Sources for NOx emissions include power plants, motor vehicles, and other sources of high-heat combustion, and sources of VOC emissions include motor vehicles, chemical plants, refineries, factories, gas stations, paint, and other sources [3].

In 2008, EPA tightened the National Ambient Air Quality Standard (NAAQS) for ozone from 80 to 75 parts per billion (ppb), calculated based on the fourth-highest daily maximum 8-h concentration across all regulatory ozone monitors in a given region, averaged across 3 consecutive years [4]. The standard was tightened again in 2015, with the new standard set at 70 ppb, though the calculation method was not changed. This 2015 standard was reaffirmed in 2020.

Importantly, the 2008 standard was not revoked after promulgation of the 2015 standard. The US EPA gives regions several years to meet each standard, and as a result, a region can be in nonattainment of the 2008 standard but in attainment of the 2015 standard even when the 2015 standard is tighter. These counter-intuitive designations that stem from the timing of standards also apply to each specific stage of nonattainment (for example, marginal, moderate, and serious). That is why, as of 2020, the Denver Metro North Front Range (DMNFR) was in serious nonattainment of the 2008 standard but only in marginal nonattainment of the 2015 standard [5, 6]. Because a designation of serious nonattainment has substantial economic consequences for a region, the older, looser standard is of most immediate concern to policymakers, even though the newer, tighter standard is more relevant to public health.

Colorado’s Front Range has struggled with ground-level ozone pollution at least since systematic monitoring began in the 1980s, presenting health risks to the region’s residents. In Colorado’s Denver-Aurora-Lakewood area, over the last three decades there have been at least 15 days per year in which ground-level ozone concentrations have been unhealthy for sensitive populations [7] with annual totals reaching as high as 81 days. Consequently, the DMNFR has consistently had difficulty meeting its obligations under the Clean Air Act.

Several studies have estimated the contribution of local factors to these high ground-level ozone values in Colorado’s Front Range. For example, Cheadle et al. found that the highest concentrations of ozone were associated with as much as 30 ppb from precursor emissions primarily from local oil and gas activity, moderate urban sources, and low agricultural sources [8].

Regional and global factors also contribute to ground-level ozone in Colorado’s Front Range. Biomass burning from agricultural practices, prescribed burns or wildfires can produce regional ozone precursor emissions. Lindaas et al. measured up to 10 ppb ozone at Boulder Atmospheric Observatory (Erie CO) during days when atmospheric transport of precursor emissions from Canada and Pacific Northwest wildfires occurred [9]. Huang et al. found interstate transport from southern California in May 2010 contributed around 1 and 15 ppb ozone on days with surface ozone levels of 60 and 75 ppb in Arizona and New Mexico, respectively, over a 1-to-3-day time period, reaching Colorado on the third day [10]. Ozone precursor emissions transported to the region from Asia have decreased as pollution controls in Asia have improved [11]. The highest non-anthropogenic production of ozone occurs during the late winter and early spring in mid-latitudes from the stratosphere transfer into the troposphere, with estimates in the western US of 17–40 ppb. This process is not significant during the summertime [1]. Springtime stratospheric intrusion episodes have been shown to be more frequent in the western US after strong La Niña winters [12].

However, weather further contributes to ground-level ozone formation in Colorado’s Front Range, with higher values typically occurring during the summer amidst higher temperatures and more hours of sunlight. Low wind and sunny summer days are the most conducive for surface ozone production in the Denver region [13]. On days when the wind is not blowing in from the west, especially the stagnant summer days, ozone precursor emissions from the east can get trapped in the region adjacent to the foothills of the Rocky Mountains [14].

As weather is a factor influencing surface ozone concentrations, it follows that climate change influences surface ozone concentration as well. Researchers have increasingly looked at the consequences of climate change on surface ozone pollution, using the term “climate penalty” to describe the additional ozone associated with climate change [15]. Recognizing the influence of temperature for catalyzing chemical reactions, some researchers have found a climate penalty factor ranging between 1 and 3 ppb ozone per degree Celsius change [15, 16].

Typically, these climate penalty approaches focus on future climate change. However, for local jurisdictions with unhealthy ozone levels, assessing the contribution of a historical climate penalty could factor into planning decisions. This is because in many locations, a global climate penalty of a few ppb could push communities into nonattainment, requiring those communities to make investments to solve the problem and protect local residents. Put another way, any region in the world that has people exposed to unhealthy ground-level ozone would benefit from information on the various contributors in order to evaluate where to deploy resources to improve public health.

In this work, we estimate the ozone climate penalty due to climatic changes between the 1950s and 2010s in Colorado’s Front Range, the period over which human-attributed climate change accelerated most dramatically [17]. We define the climate penalty as the difference between ozone concentrations observed in the 2010s and predicted ozone concentrations under a counterfactual scenario based on the 2010s but with daily weather adjusted to match the observed seasonal climate trends in 1950s. We estimated this penalty over all warm season (May–October) days in the 2010s and over a dense grid of locations. To calculate the penalty, we used spatio-temporal land-use regression (LUR) models incorporating weather, elevation, population density, and traffic variables. We used changes in seasonal and spatial trends of four local weather variables (maximum daily temperature, mean daily wind speed, relative humidity, and sea level pressure) between the two decades as representative of the changes in climate that the region experienced during that time frame. Based on this analysis, we also estimated the impact of this climate penalty on regional attainment of the EPA ozone NAAQS and assessed which sociodemographic groups and groups with chronic health conditions were associated with a higher climate penalty.

This analysis helps identify which populations on the Front Range could have benefited most from earlier action to reduce global climate change. Regional efforts to reduce ground-level ozone in the Colorado Front-Range region have successfully decreased levels of the pollutant, but levels still often exceed NAAQS limits during the summer months. When out of compliance with regulations, every part per billion ozone climate penalty can have significant health and economic implications for affected communities.

## Methods

### Study region and ground-based monitors

**Fig. 1:**
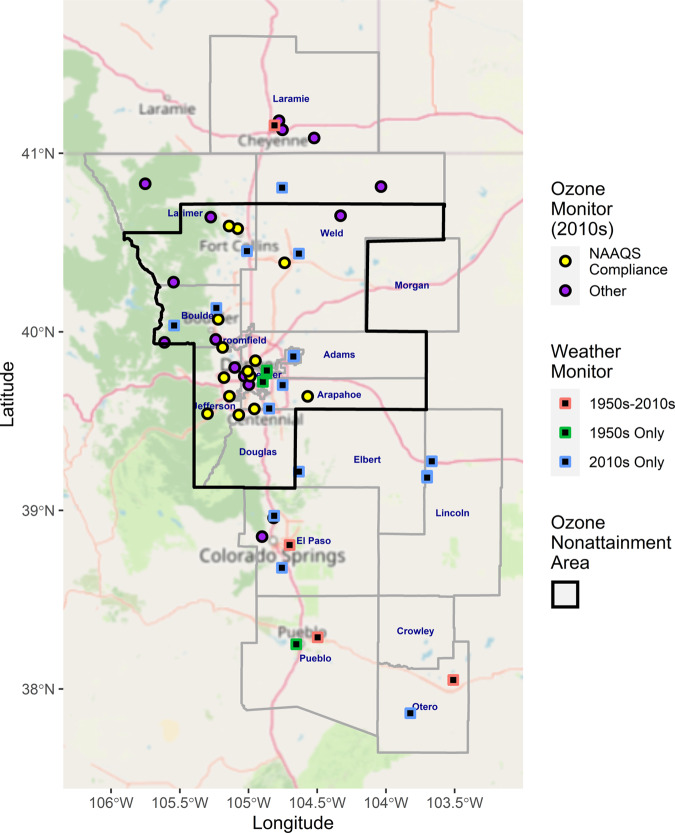
Study region and monitor sites. The study region includes 17 counties, one in Wyoming and the remainder in Colorado. The outlined region is the Metro Denver North Front Range ozone 2008 NAAQS nonattainment region.

The study region encompassed the counties of the Front Range urban corridor between Laramie, Wyoming and Pueblo, Colorado along Interstate Highway 25 (I-25), as well as more sparsely populated rural counties on the plains to the east (Fig. 1). Counties to the west were not included because the mountains of the Front Range put them in a distinct airshed. The region includes 17 counties, all but one of which are in Colorado. The US EPA NAAQS ozone nonattainment area is shown outlined in black in Fig. 1 and includes the city of Denver and its suburbs as well as the cities of Boulder, Fort Collins, and Greeley.

This region includes 21 weather monitoring sites (Fig. 1). However, as our study spans the 1950s and the 2010s, not all sites were active in all study years. In particular, three sites had monitors that were active in the 1950s but not the 2010s, an additional four sites were active in both decades, and the remaining sites were active only in the 2010s.

Daily data from these monitors were downloaded from the National Oceanic and Atmospheric Administration’s National Centers for Environmental Information and included: mean, maximum, minimum, and dew point temperature; station and sea level pressure; mean and maximum sustained wind speed; wind gust speed; precipitation and snow depth [18]. We calculated relative humidity from the mean daily and dew point temperatures. Based on data completeness, collinearity between related variables, and known relationships to ozone, we focused our analysis on four of these daily variables: maximum temperature, mean wind speed, relative humidity, and sea level pressure.

The region also includes 26 ozone monitoring sites (Fig. 1). Of these, 14 were used by the Colorado Department of Public Health and Environment to assess NAAQS attainment during the 2010s. All of the latter sites fall within the NAAQS nonattainment area. Because the decade of the 1950s predates the passage of the Clean Air Act of 1970 and the creation of the US EPA, no ozone data from the 1950s are available for the study region. Daily 8-h maximum ozone mixing ratios from all 26 ozone monitors were downloaded from the US EPA Air Quality System [19].

Because ozone usually only reaches high concentrations during warmer months, we limited our study to the 6 warmest months of each year, May through October [1, 4].

Other geospatial variables crucial for modeling ozone and weather included elevation, population density, and traffic density. Elevations of these points were evaluated using the elevatr package (version 0.2.0) [20]. Population density data from 2015 at 30 arc-second resolution were provided by Columbia University’s Socioeconomic Data and Applications Center’s Gridded Population of the World version 4 [21]. Highway Vehicle Miles Traveled shapefiles for Colorado and Wyoming in the years 2011–2017 were provided by the US Department of Transportation Highway Performance Management System [22]. Maps of these variables are given in Supplementary Fig. S1.

### Spatio-temporal land-use regressions

Our goal was to estimate the spatially resolved and temporally resolved ozone climate penalty on the Front Range. To do so, we compared spatio-temporal ozone fields in the 2010s (“Observed 2010s”) to counterfactual ozone fields that would have been observed had the daily 2010s meteorology been shifted to align with a 1950s climate (“Counterfactual 1950s”). These ozone fields and the weather fields influencing them were developed using spatio-temporal LUR models. These models do not directly characterize the underlying physical, chemical, or biological processes influencing ozone formation, but instead infer statistical relationships between empirical ozone and weather observations and other environmental variables varying in space and/or time. These statistical relationships are then used to predict ozone or weather variables at unmonitored locations or times.

All modeling was performed in the R language (version 4.0.2) with the mgcv package (version 1.8-31) [23–25]. Mean daily wind speed was log-transformed, and the relative humidity, expressed as a value on the unit interval, was probit-transformed. Computing the ozone climate penalty required several steps, which are visualized in Supplementary Fig. S2 and described below:

Step 1: For each of the four meteorological variables, we fit a spatio-temporal LUR model to the 1950s and 2010s together. Specifically, we used a generalized additive mixed model (GAMM) fitted to daily meteorology monitor observations with fixed effects of spatially indexed variables and a smooth seasonal trend (up to 6 degrees of freedom per year; possibly crossed with the spatially index variables), and a random effect by date. The full mixed effects models captured daily weather conditions across the DMNFR in each decade, while the fixed effects portion of the model captured the decade-specific spatial and seasonal climate trends. That is, the full model characterized the weather while the fixed effects portion characterized the climate. The final set of fixed and random effects was chosen independently for each meteorological variable using leave-one-monitor-out cross-validation and is listed in Supplementary Table S1.

Step 2: For each meteorological variable, we used the corresponding LUR to perform spatial prediction to estimate the daily values of the weather and climate fields on a dense grid over the study domain. This grid had a resolution of 1/48 degree in longitude and 1/36 degree in latitude. We also predicted at each ozone monitor location.

Step 3: For each meteorological variable, we calculated a counterfactual 1950s weather field by taking the observed 2010s weather field and subtracting the difference between the 2010s and 1950s climate fields at each grid point and ozone monitor location on each day.

Step 4: We created an ozone LUR to model the relationship between ozone and meteorology over the 2010s. Specifically, we fit the observed daily 8-h maximum ozone monitor observations using a GAMM with fixed effects selected from among the (potentially nonlinear functions of the) weather fields, latitude, longitude, elevation, log10 population density, log10 inverse-distance weighted traffic density, as well as categorical year and smooth trends over location and date. The non-meteorological variables in the fixed and random terms attempted to capture non-meteorological factors influencing ozone production, such as ambient NOx and VOC concentrations. The final model, selected through leave-one-monitor-out cross-validation, included a five-way interaction between the four weather fields and categorical year, separate additive effects of population density and traffic, a smooth trend over spatial location (two degrees of freedom), a smooth trend over date (six degrees of freedom per year), and an interaction between location and day of the year. The random effects included separate coefficients for the intercept, latitude, longitude, and elevation on each date. These random effects represented the aggregate of all non-meteorological time-varying influences on ozone concentrations.

Step 5: Using the ozone LUR, we performed spatial prediction to estimate the daily 8-h maximum ozone concentration field at each grid location, each ozone monitor location, and at the centroid of each of the 1003 census tracts in the domain. This procedure yielded our observed 2010s ozone field.

Step 6: We fed the counterfactual 1950s weather fields into the ozone LUR to predict what the 2010s daily ozone concentration field would have been had the meteorology been different, but all other factors held constant. This procedure yielded our counterfactual 1950s ozone field.

Step 7: Finally, we calculated the ozone climate penalty by subtracting the counterfactual 1950s ozone field from the observed 2010s ozone field. The result was an estimate of the ozone climate penalty on each date at each spatial location referred to in Step 5.

### NAAQS attainment

Next, we evaluated how the ozone climate penalty has made attainment of the ozone NAAQS more difficult. First, we mapped the average number of annual NAAQS exceedances over our study domain under the observed 2010s ozone field and compared them to the average number of annual exceedances under the counterfactual 1950s ozone field. Second, we contrasted the true DMNFR ozone design values to the design values that would have occurred under the counterfactual 1950s climate. Design values are the numbers (computed from air pollution monitor observations) that are directly compared against the NAAQS to evaluate regional attainment. This comparison proceeded in two steps as visualized in Supplementary Fig. S3 and described here:

Step 1: We estimated counterfactual 1950s ozone observations by taking the true 2010s ozone monitor observations at the 14 monitors used to assess attainment and subtracted the difference between the observed 2010s ozone field and the counterfactual 1950s ozone field evaluated at these locations.

Step 2: We calculated the design values for the 2010s ozone observations and for the counterfactual 1950s ozone observations.

Given these design values we were able to predict the expected attainment year of the 2008 (75 ppb) and 2015 (70 ppb) standards under the 2010s climate and under a counterfactual 1950s climate. This simple extrapolation effectively assumed regulatory actions continue to push precursor emissions lower at the historical rate and temperatures do not continue increasing.

### Health equity

Because neither population characteristics nor the ozone climate penalty is spatially uniform, the penalty is greater for some populations than others. To determine which populations were associated with the greatest ozone increase in ambient ozone driven by climate change (or, conversely, which populations could have benefited most from earlier climate action), we estimated the ozone climate penalty at each census tract. For tracts large enough to overlap three or more points from our prediction grid, we used a population-weighted approached to estimate this penalty. For tracts overlapping two or fewer grid points, we evaluated the penalty at the tract centroid. We then regressed the penalties at all 1003 tracts against tract-level sociodemographic and health burden variables from the 2018 American Community Survey 5-year estimates and from the Colorado Department of Public Health and Environment. Specifically, we tested race, Hispanic ethnicity, foreign birth, child poverty, and rates of asthma, diabetes, heart disease, obesity, healthcare coverage, and overall health status. Census tracts in Laramie County, Wyoming (*n* = 7) were included in demographic analyses but were not included in the analysis of health burdens because health burden data were produced by a Colorado state agency. We also tested the difference between tracts that overlap the DMNFR ozone nonattainment area and tracts that do not. For each variable, we tested simple models as well as models controlling for urbanicity either through tract area or tract population density.

## Results

### Meteorology and climate models

Seasonal results from the climate and weather models for each meteorological variable averaged over all years in each decade and spatial grid locations in the modeling domain were plotted (Supplementary Fig. S4). The maximum daily temperature was 1.6–2.2 °C (3–4 °F) higher in the 2010s than in the 1950s during most of the warm season months; mean daily wind speed was lower in the 2010s during the spring and early summer. The seasonal trends for relative humidity and sea level pressure were similar between decades.

Spatial results from the climate and weather models for each meteorological variable were averaged over all warm season days in each decade and mapped (Supplementary Fig. S5). Elevation had a major influence on local meteorology along the Front Range; mean wind speed, sea level pressure, and maximum temperature all showed strong elevation dependence. Relative humidity displayed a nonlinear dependence on longitude and latitude.

All four variables displayed systematic differences in spatial trends between decades (Supplementary Fig. S5), though for relative humidity and sea level pressure this difference was relatively small (−0.033% for humidity, 0.0326 mb for pressure). Wind speed dropped between 0.5 and 1.5 knots from the 1950s to the 2010s with the largest drops in the high elevation areas; maximum temperature increased by 2.0 °C (3.6 °F). Because the spatially indexed variables selected by the predictive climate models for sea level pressure and maximum temperature did not vary between decades the difference surfaces are spatially uniform even though the temporal differences are not uniform over time (Supplementary Fig. S4).

### Ozone models

**Fig. 2:**
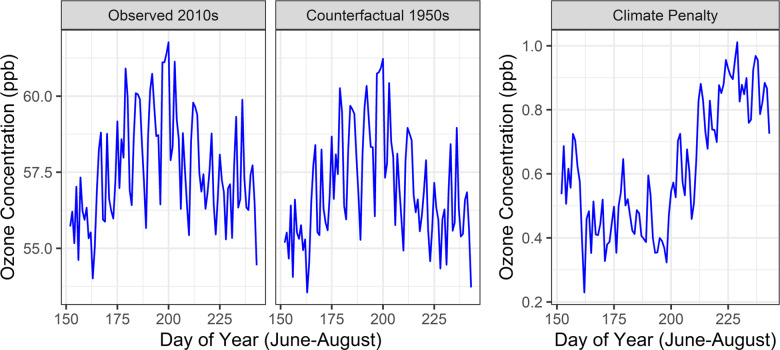
June-August daily average ozone concentrations. Ozone concentrations on each day of year during the high ozone period under the observed 2010s climate, the counterfactual 1950s climate, and their difference.

We present results focusing on the 3-month high ozone season, June–August, a subset of our full warm season time domain. Results for the full warm May–October warm season are given in the supplement. Ozone during the 2010s was highest around mid-July (200th day of the year; Fig. 2). The same was true for our counterfactual 1950s predictions. However, the difference between the two decades—the ozone climate penalty—was highest later in the summer, averaging around 0.5 ppb before the 200th day of the year but rising to roughly 1.0 ppb after and through August (Fig. 2; Supplementary Table S2). Both the overall concentration and the penalty drop in early September (Supplementary Fig. S6).

Population-weighted monthly average ozone climate penalties averaged over counties track together relatively closely over time (Supplementary Fig. S7), indicating that the temporal variation in the ozone penalty is larger than spatial variation on the scale of counties. The largest difference between the county with the highest penalty and the county with the lowest occurs in August where the range of penalties is 0.17 ppb. Thus, capturing local-scale variability in the penalty is vital for accurately estimating health impacts.

**Fig. 3:**
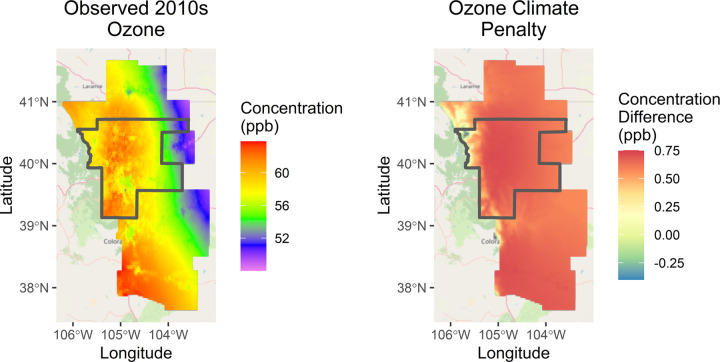
June-August average ozone concentrations and ozone climate penalty. Ozone concentrations across the modeling domain during the high ozone period (June–August) under the observed 2010s climate and the difference between the observed and counterfactual climates.

June–August Ozone displayed marked spatial patterns with gradients identifiable by population density, traffic proximity, altitude, latitude, and longitude (Fig. 3). Predicted concentrations were generally lower in proximity to roadway traffic as expected given titration of ozone by traffic-emitted NO, but higher in suburban areas surrounding the major urban centers. Our LUR also estimated higher ozone in the less-populated southern quarter of our study domain, though as shown in Fig. 1 there is no ozone monitoring in this region to constrain the model. Spatial patterns over the full warm season (May–October) display similar spatial gradients (Supplementary Fig. S8).

The ozone penalty itself is highest in the lowest elevation regions, which includes the major urban centers at the foot of the Front Range within the DMNFR nonattainment area: Boulder, Fort Collins, Greeley, and Denver and its suburbs (Figs. 1, 3, and S8). In this region, the ozone climate penalty reaches an average of 0.75 ppb per day during the high ozone months (Fig. 3).

### NAAQS attainment

**Fig. 4:**
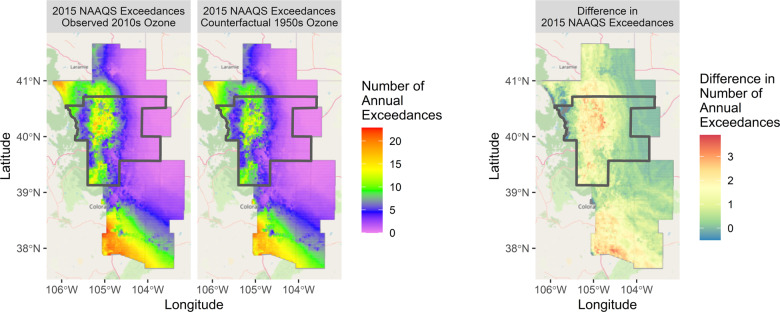
Annual average number of ozone 2015 NAAQS exceedances. (Left) The average number of annual exceedances of the 2015 ozone NAAQS (70 ppb) during the months of June–August under the observed 2010s climate and the counterfactual 1950s climate. (Right) The difference in the number of annual exceedances of the 2015 standard between the observed 2010s and counterfactual 1950s.

Figure 4 shows the average annual number of 2015 ozone NAAQS (70 ppb) exceedances at each grid point in our study domain. The I-25 urban corridor between Colorado Springs and Fort Collins displays roughly 15 exceedances per year, higher than the surrounding less-populated areas, which have none. The number of exceedances is also high in the high-altitude extreme west of our domain and in the extreme south of our domain where there is no ozone monitoring to constrain the LUR. That said because our LURs assume spatial smoothness they may underestimate the true number of local exceedances. But given this limitation, under the counterfactual 1950s climate the I-25 urban centers were found to experience approximately 3 fewer exceedance per year.

Results for the full warm season (May-October) appear nearly identical to the high ozone season (Figs. 4 and S9). Results for the 2008 standard (75 ppb) during June–August show roughly one additional exceedance per year due to the climate penalty (Supplementary Fig. S10).

**Fig. 5:**
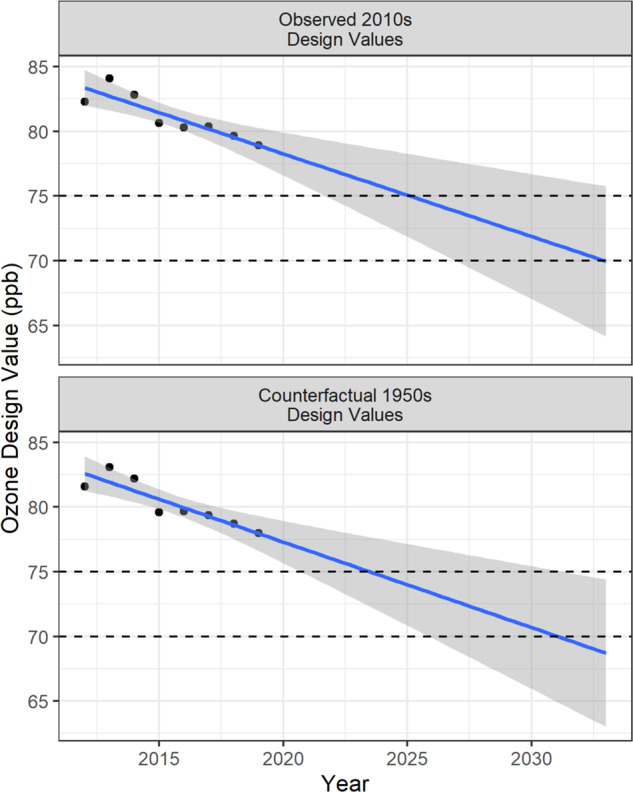
Ozone design values for the Metro Denver North Front Range. Design values based on the monitored ozone daily concentrations (left panel) and based on the monitored daily concentrations adjusted by the time- and location-specific climate penalty (right panel). Design values are based on 3-year running averages so, e.g., the 2012 design value incorporates monitor observations from 2010, 2011, and 2012. Best-fit lines and prediction error envelopes are shown in blue and gray, respectively. Dashed lines display the levels of the 75 ppb and 70 ppb standards.

Observed ozone design values (the value computed from regulatory monitor observations that is compared to the NAAQS to assess regional attainment) for the DMNFR in the 2010s were compared with the design values under the counterfactual 1950s climate (Fig. 5). Observed design values trended downward over time during the 2010s as regulatory actions decreased precursor emissions. The climate penalty was found to increase the design values by roughly 1 ppb compared to what they would have been under the counterfactual 1950s climate. Extrapolating the design value trends forward in time, the DMNFR would expect to reach attainment of the 2008 (75 ppb) standard in 2025 under the observed 2010s climate but could have reached attainment in 2023 under the counterfactual 1950s climate. A similar delay also applies to attainment of the 2015 (70 ppb) standard, which the DMNFR would expect to meet in 2035 but could have met by 2033 without the climate penalty.

### Health equity

**Fig. 6:**
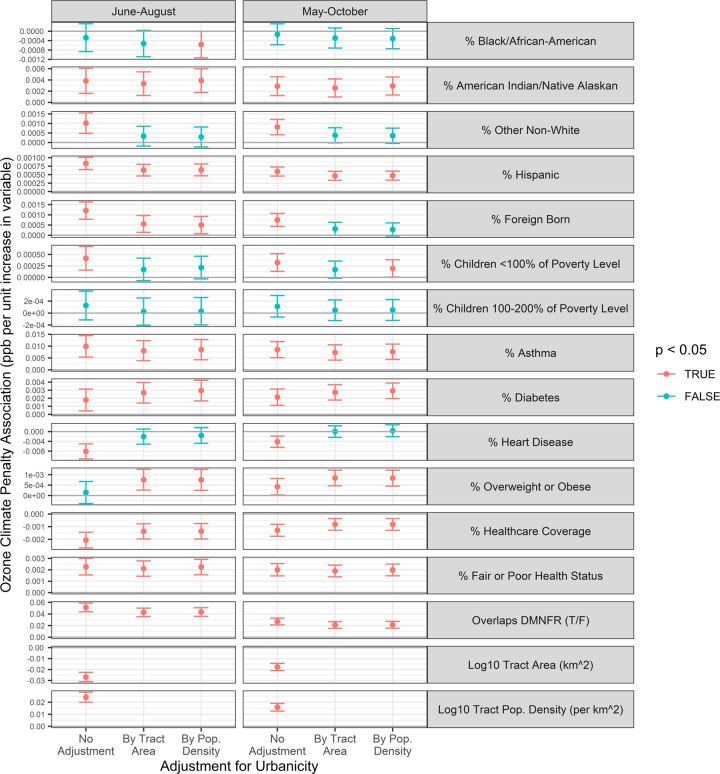
June-August and May-October ozone climate penalty health equity associations. Point estimates and 95% confidence intervals of associations between tract ozone climate penalties and tract health equity measures.

Associations between census tract population-weighted ozone climate penalties and health equity variables are shown in Fig. 6. Results are shown separately for simple univariate models and models controlling for urbanicity via tract area or population density, as well as for models using the warm season (May–October) ozone climate penalty or the high ozone season (June–August) ozone climate penalty as the outcome. Tract area and population density were negatively and positively associated, respectively, with the ozone climate penalty.

Several health equity variables were consistently found to be positively associated with the ozone climate penalty: % American Indian/Native Alaskan, % Hispanic, % asthma, % diabetes, % with fair or poor health status, and whether the tract overlaps the DMNFR nonattainment area. Only % with Healthcare Coverage was negatively associated with the ozone climate penalty. The strongest positive association with the ozone climate penalty was found with % Asthma, where a 1 percentage point increase in the tract asthma rate as associated with a 0.010 ppb higher ozone climate penalty on each day of the high ozone season. However, multiplying each association by the standard deviation of the health equity predictor variable, i.e., normalizing across the observed variability across tracts, showed that whether the tract overlaps the DMNFR nonattainment area was the strongest predictor, followed distantly by % with healthcare coverage and % with fair or poor health status (Supplementary Fig. S11).

## Discussion

Our findings of a modest ozone climate penalty on the Front Range are broadly consistent with estimates of several ppb daily maximum 8-h surface ozone per degree Celsius temperature change [26]. The ozone climate penalty can be expected to grow over the next several decades. The Earth’s surface will continue to absorb heat trapped by long-lived greenhouse gases emitted over the past two centuries [27]. Additionally, even under the most optimistic climate mitigation scenarios, the world will continue to add carbon to the atmosphere for two more decades [28] along with associated precursor emissions for ozone formation. Without substantial intervention, warming is virtually guaranteed for the decades to come, and with it, difficulty controlling ground-level ozone.

While changes in climate affect everyone, people living in certain geographic areas are at heightened risk of experiencing adverse health outcomes worsened by climate change. Rising temperatures can exacerbate existing health conditions, including respiratory illnesses such as asthma and COPD. An increase in temperature raises the level of ozone and other pollutants in the air, exacerbating some health conditions, including premature deaths, acute and chronic cardiovascular and respiratory illnesses [29, 30].

Even modest changes to long-term ozone levels can have substantial impacts on respiratory health, especially in children. For example, Lin et al. found that a 1 ppb increase in 8-h max ozone over a summer ozone season was associated with a 22% higher odds of pediatric asthma hospitalization in New York State [31], while Hwang et al. found that this exposure was associated with a 4–6 mL decrement in pediatric lung function growth (FVC, FEV_1_, and FEF_25-75_) in Taiwan [32].

We leave the investigation of the ozone climate penalty’s full health impact to future work. However, at the census tract level, the penalty was consistently and positively associated with the percentage of Hispanics living in that tract, the percentage of children living within 100–200% of the federal poverty level, and the percentage of residents with asthma, diabetes, fair or poor health status, or lacking health insurance. Thus, the ozone-specific health burden due to climate change since the 1950s has fallen disproportionately on disenfranchised and frontline communities. Conversely, these communities could have benefited the most from past climate action and could benefit the most from future mitigation. Indeed, given the association between the penalty and percent with asthma, a disease that is directly exacerbated by ozone, future climate action that reduces ozone on the Front Range could have a particularly positive impact on this patient group.

Furthermore, communities with the highest minority and low-income populations would also be expected to benefit most directly from actions to lower ozone precursor emissions and their associated co-pollutants because these communities are often located near major sources; Many of the precursor and co-pollutant gases are themselves harmful. For example, the majority-Hispanic residents of Denver’s Globeville-Elyria-Swansea neighborhood cluster live in close proximity to three interstate highways, numerous marijuana grow operations [33], a power plant, and the state’s largest oil and gas refinery. Community organizers have asked the state to revoke the refinery’s operating license over concerns about hydrogen cyanide and VOC emissions [34]. Doing so could provide both short-term and long-term health benefits to its neighbors.

The association with the percentage of Hispanic community members indicates the burden imposed by the ozone climate penalty is skewed along racial and ethnic lines, potentially reinforcing disparities in housing, employment, and education as well as in exposures to other environmental stressors such as noise, diesel particles, and the ozone precursors themselves. The percentage of Black/African-American community members was not consistently associated with the climate penalty. This result is due to the fact that Black/African-American community members are highly concentrated in Colorado’s urban centers where the climate penalty was estimated to be relatively uniform. However, given that the climate penalty is high precisely in these urban areas, we would expect people living there to benefit from climate action as well.

In addition to the adverse health outcomes, two additional years of NAAQS nonattainment have substantial economic consequences for the State of Colorado and municipalities in the region.

This work had several strengths: First, it selects the best-predicting LURs from among a comprehensive family of possible inferential models. Second, it studies the ozone climate penalty on a scale relevant to local, regional, and national policymaking. Third, it quantifies the ozone climate penalty at individual ozone monitor sites such that its impact on ozone design values and thus NAAQS attainment can be quantified. Fourth, its spatial resolution enables determination of which census tracts (and thus which Front Range sociodemographic groups) were most impacted by warming-attributed ozone.

This work also has several limitations. First, it assumes a degree of spatial smoothness in the ozone fields and thus could miss important local-scale processes influencing ambient ozone levels. However, since ozone is a secondary pollutant, smoothness is likely a reasonable approximation. Second, the work does not attempt to model physical, chemical, or biological processes directly, but instead relies on those processes being implicitly captured by empirical monitor data. Thus, if certain processes or feedbacks are only significant in areas that happen not to have ozone monitors, the LUR’s predictions in those areas may not be accurate. However, in the DMNFR, areas without ozone monitoring are not well-populated and (by construction) do not inform NAAQS attainment.

Given that many ozone precursor gases are emitted during fossil fuel extraction, transportation, processing, and/or burning, and thus are inextricably linked to anthropogenic climate warming, well-designed regulatory actions that decrease ozone precursor emissions have the potential to limit ozone formation in two ways: directly through lower precursor concentrations and indirectly through slower climate warming.

However, global climate change and associated projected increases in the extreme heat that leads to ozone formation will make achieving and remaining in attainment increasingly difficult. If moderate action is taken to reduce greenhouse gas emissions (RCP4.5), significant increases in the number of days with temperatures above high thresholds are expected even at midcentury across the case study region [35]. For example, in Boulder County, while historically (1971–2000) there have been on average 2 days per year with a heat index above 32.2 °C (90 °F), this threshold is projected to be exceeded 12 times in an average year by midcentury [18]. Under a higher emissions scenario (RCP8.5), this would increase to 17 exceedances per year. In Morgan County, where there have been 15 days on average with a heat index above 32.2 °C (90 °F), under RCP4.5 this would increase to 45 by midcentury and to 55 under RCP8.5.

Importantly, we found that climate change is already contributing significantly to the DMNFR’s nonattainment status and is delaying projected attainment of the 2008 and 2015 standards by around 2 years each. Information that disentangles the causes of ground-level ozone pollution is critical for communities experiencing its adverse effects. This information can empower disproportionately affected communities in their pursuit of both preventing future harms and acquiring resources needed to deal with those harms that have already occurred, or those that are unavoidable in the future due to past greenhouse gas emissions. Yet, localized information on climate change’s role in ground-level ozone pollution has not been readily available to date. The approach pursued here takes existing methods a step further so that such local analyses can be conducted to help inform community and local-to-regional planning decisions.

In summary, we found that residents of the Front Range are already affected by climate change through higher temperatures and higher ozone levels, and that the resulting ozone burden is already falling disproportionately on historically disenfranchised and frontline communities. Thus, our results underscore the need for aggressive climate mitigation and adaptation action that centers those communities.

## Supplementary information

Supplementary information

## Data Availability

Data generated for this manuscript can be found at the OSF repository: https://osf.io/ynv4e/.
