# Supplementary material for: The ozone climate penalty, NAAQS attainment, and health equity along the Colorado Front Range
Source: J Expo Sci Environ Epidemiol. 2021 Sep 10;32(4):545–53. doi: 10.1038/s41370-021-00375-9 (PMC9349035; doi:10.1038/s41370-021-00375-9)
Supplement: Supplementary file 1 — Supplementary information [file 41370_2021_375_MOESM1_ESM.docx]

**Supplemental File – Crooks Et Al.**


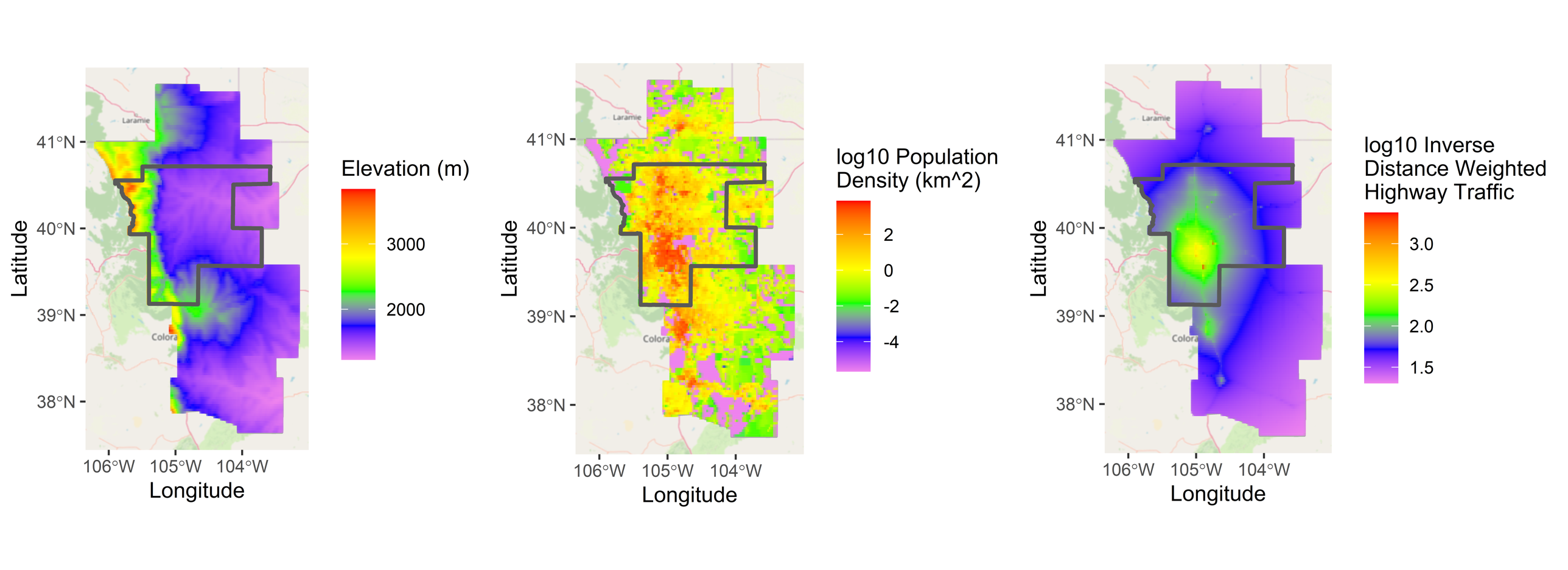


Supplemental Figure S1: Maps of spatial variables used in predictive modeling


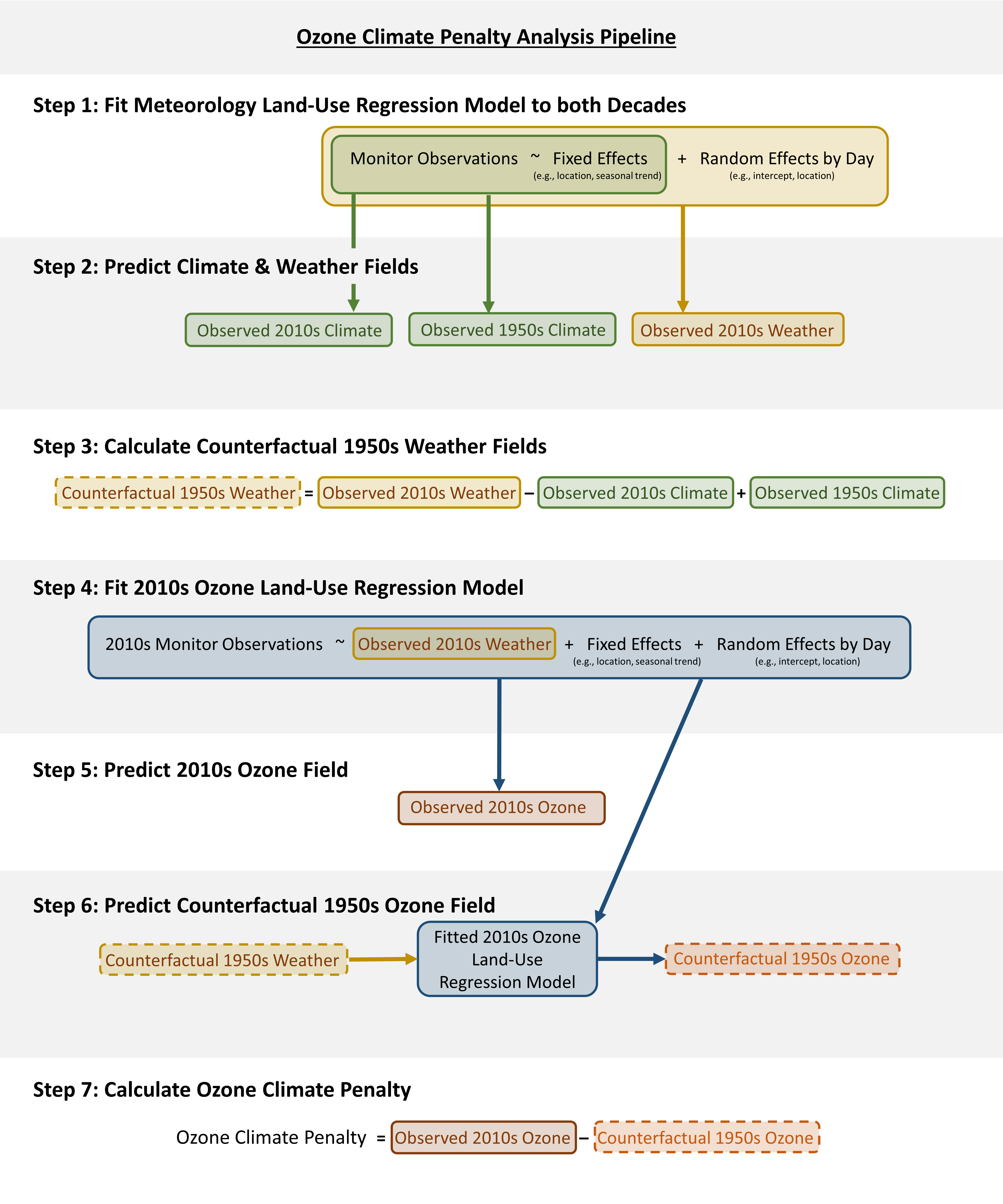


Supplemental Figure S2: Visual representation of the data analysis pipeline for calculating the ozone climate penalty.


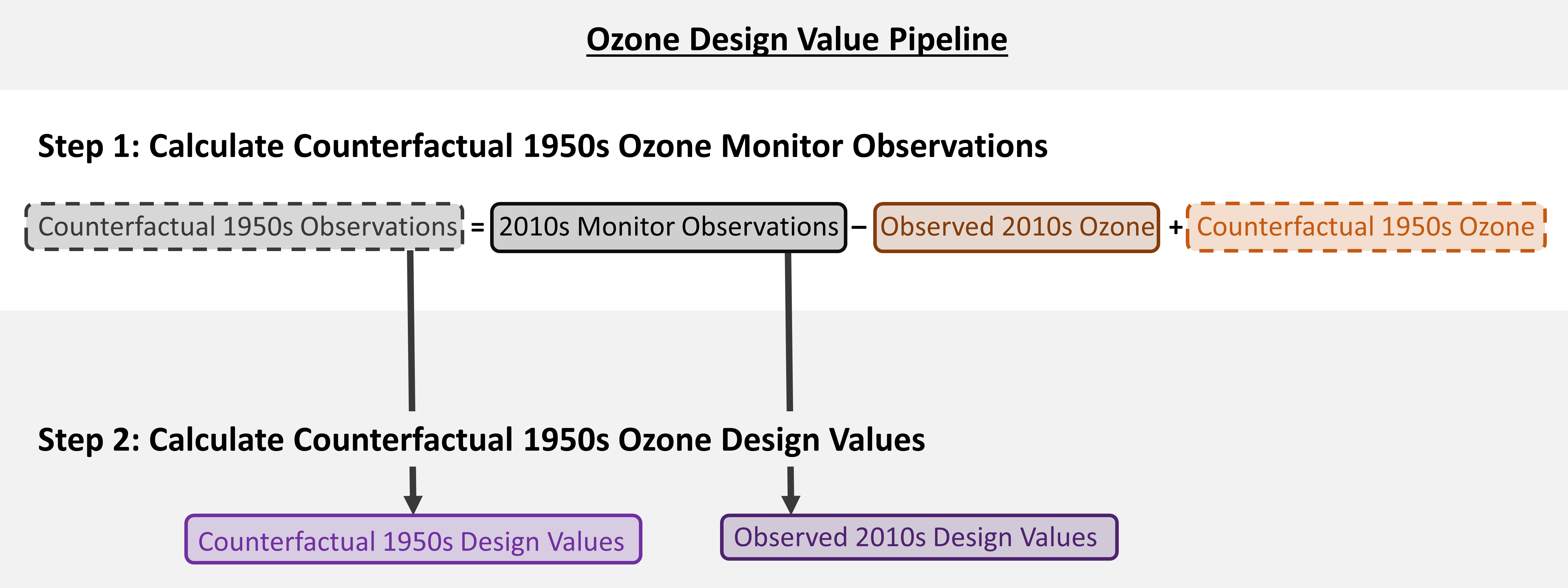


Supplemental Figure S3: Visual representation of the analysis pipeline for computing true and counterfactual ozone design values


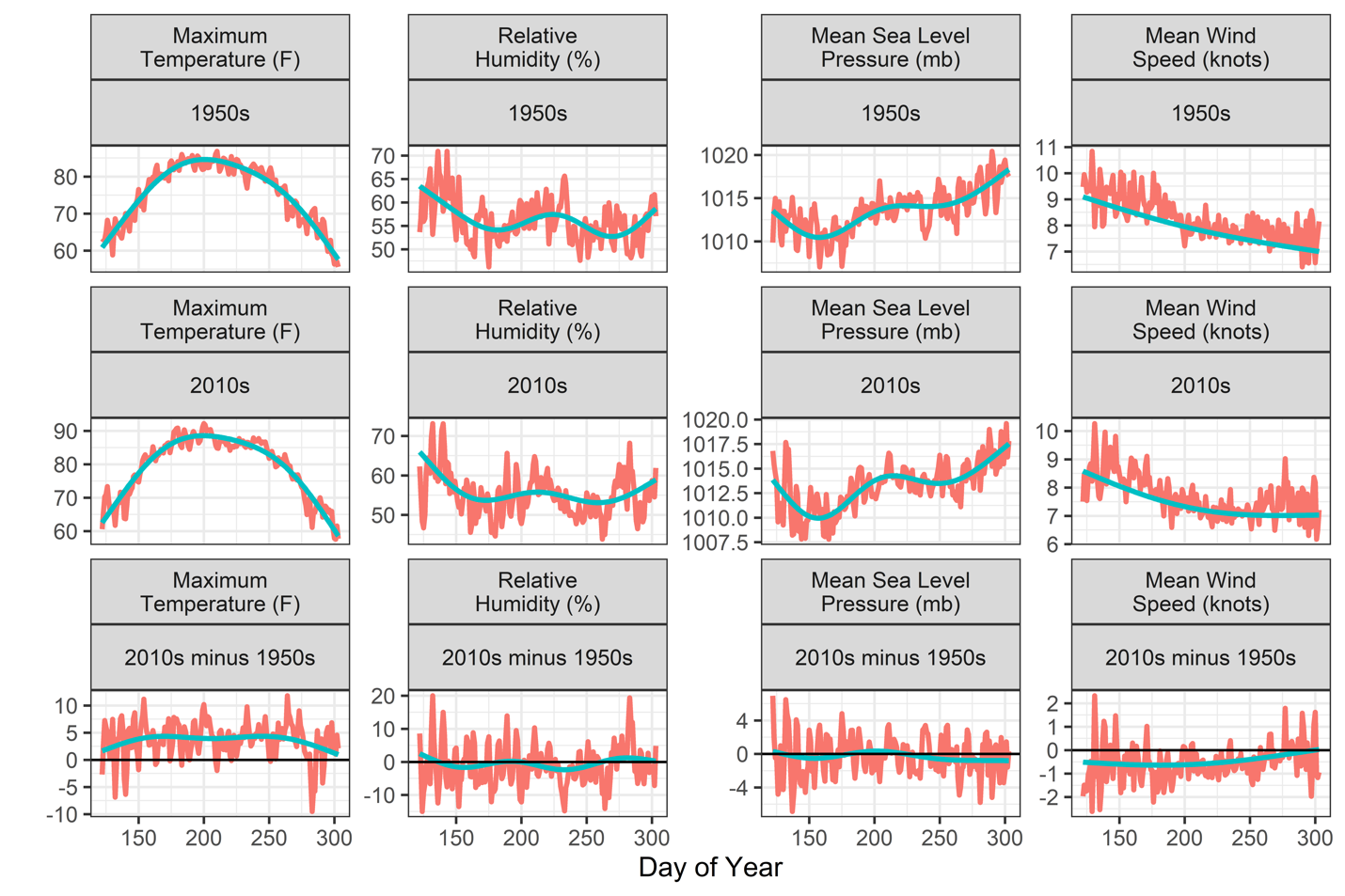


Supplemental Figure S4: Estimated warm month (May-October) seasonal and daily trends in meteorological variables in the 1950s (top row) and 2010s (middle row) and their difference (bottom row). Results from weather models are shown in red (averaged over all years in each decade). Results from climate models are shown in blue.


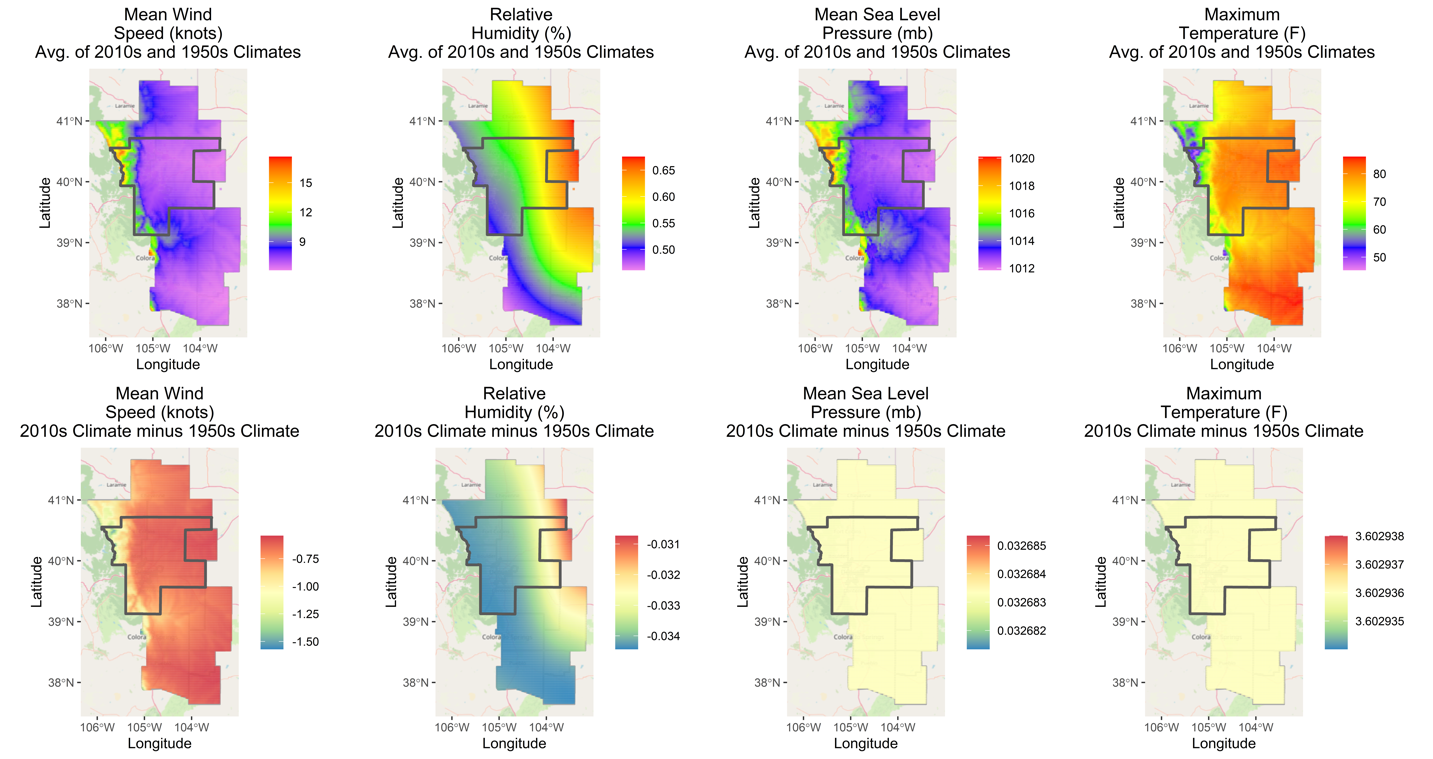


Supplemental Figure S5: Estimated warm month spatial trends in meteorological variables including the average of the decade-specific trends (top row) and difference between them (bottom row).


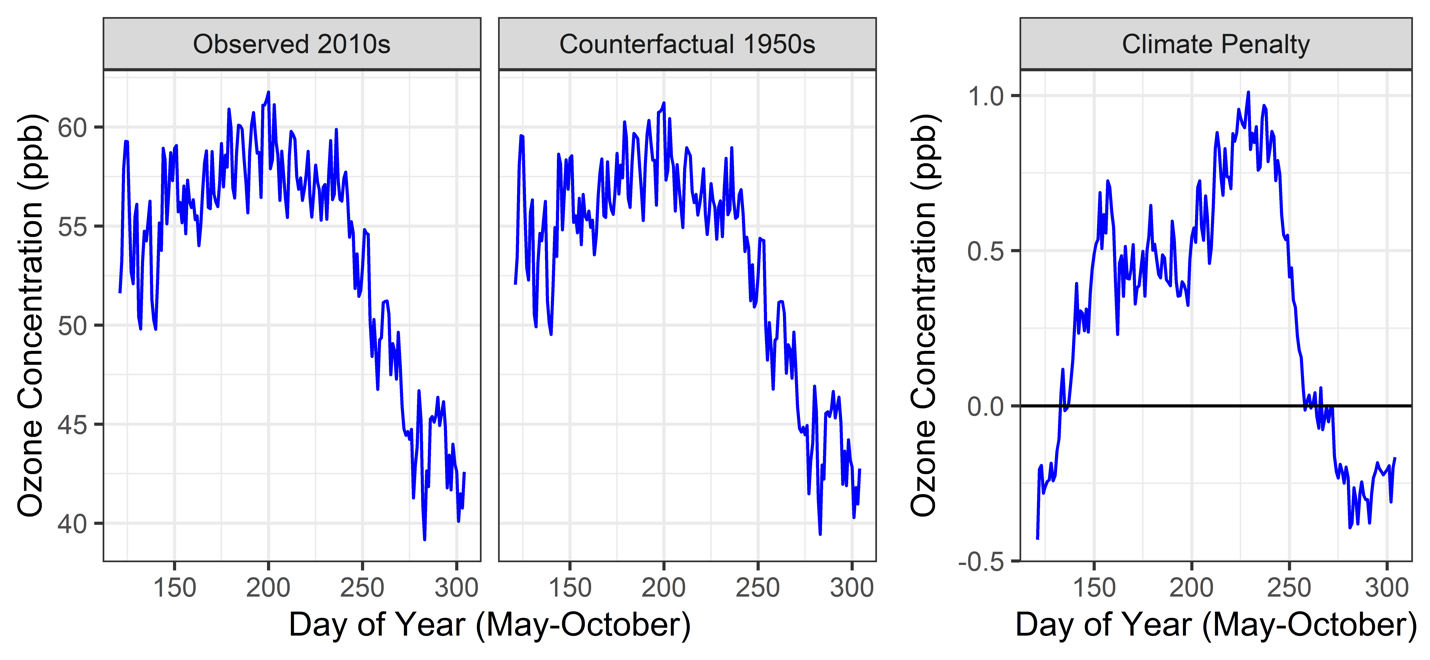


Supplemental Figure S6: Ozone concentrations on each day of year during the high ozone period averaged by decade under the observed 2010s climate, the counterfactual 1950s climate, and their difference.


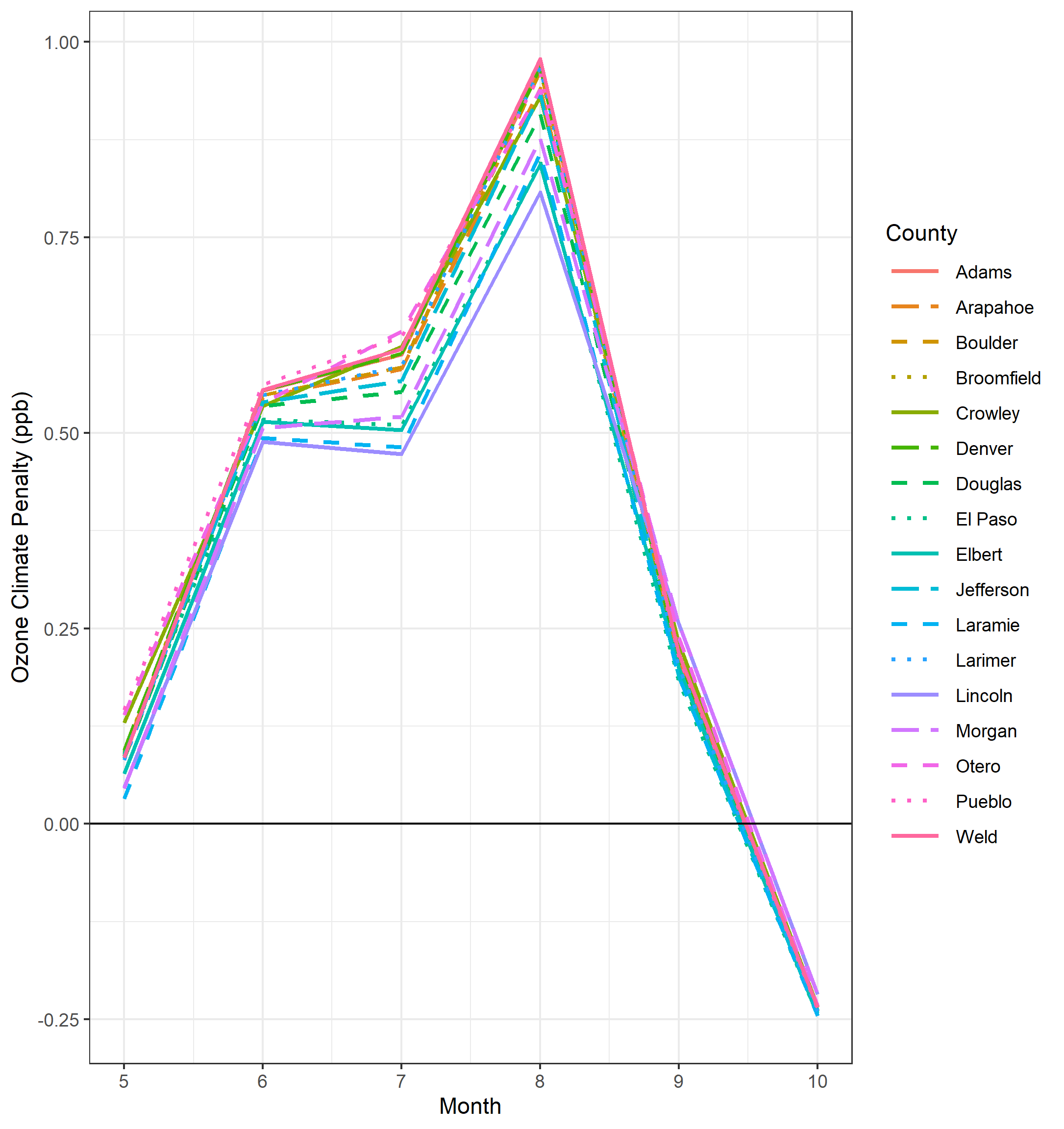


Supplemental Figure S7: Monthly average ozone climate penalties by county


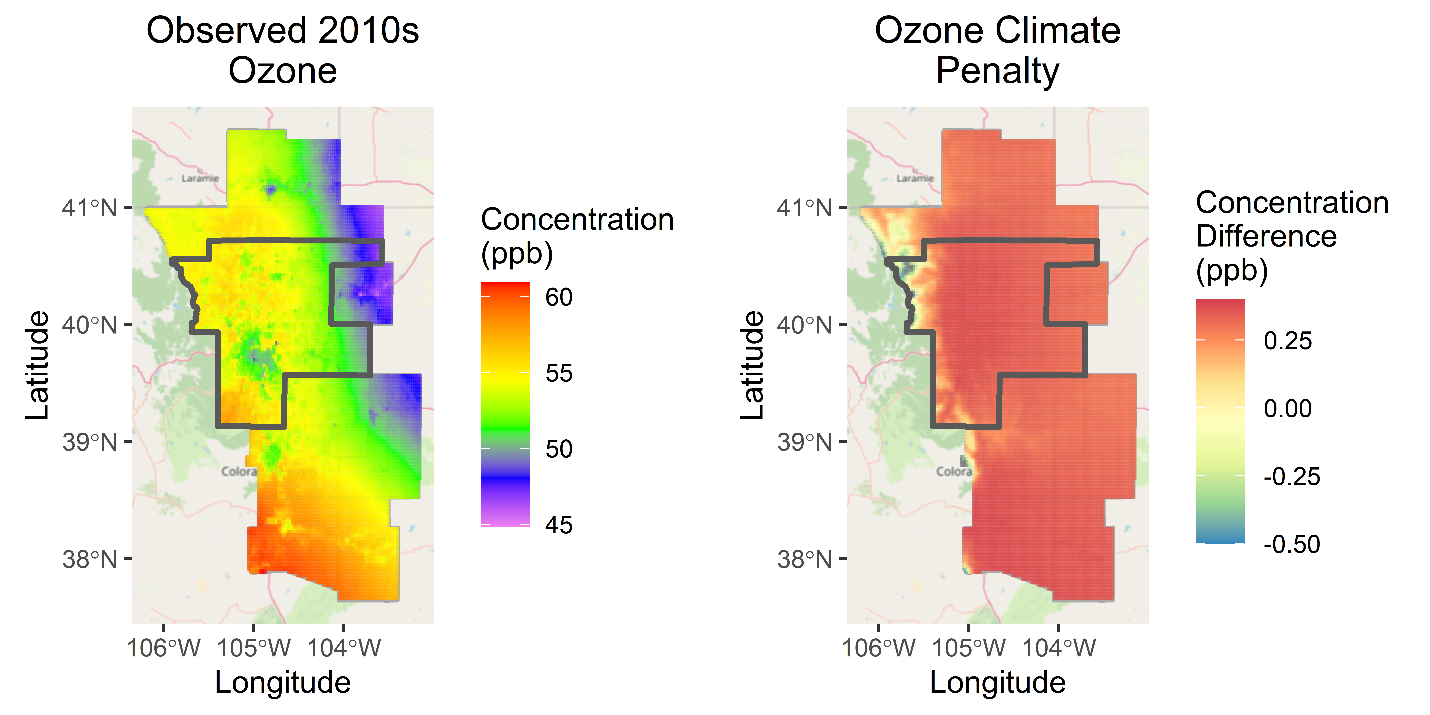


Supplemental Figure S8: Ozone concentrations across the modeling domain during the warm season (May-October) averaged by decade under the observed 2010s climate and the difference between the observed 2010s and the counterfactual 1950s climates (the climate penalty)


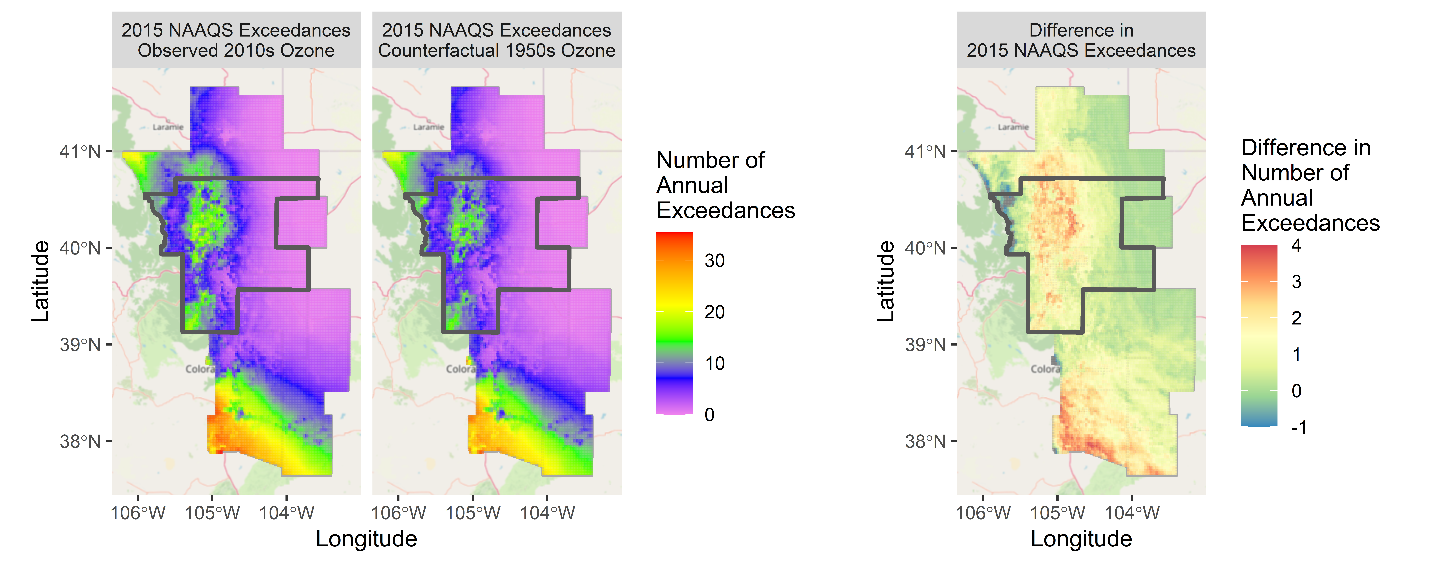


Supplemental Figure S9: (left) The average number of annual exceedances of the 2015 ozone NAAQS (70 ppb) during the months of May-October under the observed 2010s climate and the counterfactual 1950s climate. (right) The difference in the number of annual exceedances of the 2015 standard between the observed 2010s and counterfactual 1950s.


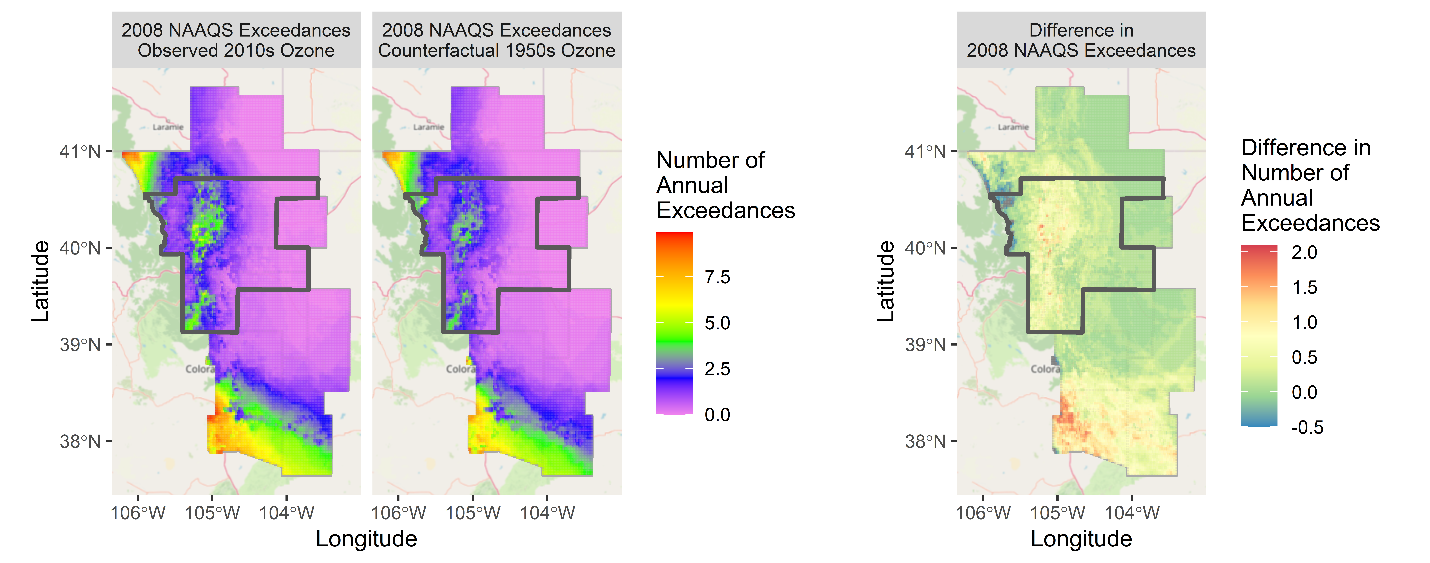


Supplemental Figure S10: (left) The average number of annual exceedances of the 2008 ozone NAAQS (75 ppb) during the months of June-August under the observed 2010s climate and the counterfactual 1950s climate. (right) The difference in the number of annual exceedances of the 2008 standard between the observed 2010s and counterfactual 1950s.





Supplemental Figure S11: Point estimates and 95% confidence intervals of associations between tract ozone climate penalties and tract health equity measures. Estimates have been rescaled by the standard deviation of the health equity variable to facilitate comparison of effect magnitude between them.

| **Variable** | **Climate LUR Model**  **(Fixed Effects)** | **Weather LUR Model**  **(Random Effects by Date)** |
| --- | --- | --- |
| Wind speed | Elevation + s(DayOfYear,2) | Intercept + longitude |
| Relative humidity | s(Location,6) + s(DayOfYear,6) + ti(Location,DayOfYear,6,6) | Intercept + Location |
| Sea level pressure | Elevation + log_10_(PopDensity) + s(DayOfYear,6) | Intercept + Location |
| Maximum temperature | Elevation + s(Location,3) + s(DayOfYear,6) + ti(Location,DayOfYear,6,6) | Intercept |

*Supplemental Table S1: Final climate and weather models for the four meteorological variables. s() denotes a thin-plate-spline smooth term. ti() denotes an interaction cross-term between two smooth terms. Numbers in parentheses indicate degrees of freedom. "Location" indicates that both latitude and longitude are present.*

| Year | May | June | July | August | September | October |
| --- | --- | --- | --- | --- | --- | --- |
| 2010 | 0.060886 | 0.666817 | 0.598728 | 0.88017 | 0.237872 | -0.23743 |
| 2011 | -0.13265 | 0.68666 | 0.697993 | 1.192148 | 0.30851 | -0.2443 |
| 2012 | -0.00792 | 0.155304 | 0.334133 | 0.515407 | 0.024774 | -0.3288 |
| 2013 | 0.118372 | 0.443613 | 0.378875 | 0.986675 | 0.18395 | -0.06333 |
| 2014 | 0.047191 | -0.03558 | 0.067954 | 0.340035 | -0.1515 | -0.42611 |
| 2015 | 0.145308 | 1.215451 | 1.214941 | 1.482302 | 0.63105 | -0.08117 |
| 2016 | -0.38987 | 0.212524 | -0.12477 | 0.42531 | -0.21966 | -0.53884 |
| 2017 | 0.120143 | 0.450174 | 0.519927 | 0.875293 | 0.311589 | -0.19889 |
| 2018 | 0.307587 | 0.498368 | 0.777082 | 0.934619 | 0.200226 | -0.22742 |
| 2019 | 0.278651 | 0.653507 | 0.551433 | 0.852258 | 0.34218 | -0.19541 |

Supplemental Table S2: The ozone climate penalty (observed 2010s minus counterfactual 1950s) by year and month.
